# Functional, textural, and rheological properties of mixed casein micelle and pea protein isolate co-dispersions

**DOI:** 10.3168/jdsc.2021-0157

**Published:** 2022-02-10

**Authors:** Abigail Krentz, Israel García-Cano, Rafael Jiménez-Flores

**Affiliations:** Department of Food Science and Technology, The Ohio State University, Parker Food Science and Technology Building, 2015 Fyffe Rd., Columbus 43210

## Abstract

In the midst of rising consumer health and environmental concerns, pea protein has increased in popularity as an alternative to animal-origin proteins. However, the use of pea protein in food systems is largely hindered by its poor functionality, including low solubility. The objective of this study was to measure the textural, functional, and rheological properties of a mixed plant- and animal-based protein system. Caseins, the major protein in bovine milk, are a known animal-based protein with optimal functional properties and high sensory acceptability. Through cold-temperature homogenization, insoluble pea proteins were incorporated with casein micelles in a stable, mixed, colloidal dispersion. Three blends with various casein-to-pea ratios (90:10, 80:20, 50:50) were prepared and analyzed. We hypothesized that incorporation with casein micelles would improve the poor functional properties of pea protein, and thus increase its potential uses in the food industry as a functional ingredient. The protein blend successfully underwent chymosin coagulation, a key ability of caseins, and formed protein gels with textures similar to commercial queso fresco and hard tofu. The 50% casein micelle:50% pea protein blend had better emulsification properties than pea protein alone. In contrast, this blend had the same foaming properties as pea protein alone. The mixed protein blends had similar rheological properties to skim milk, thus increasing their potential applications in the food industry. These results serve as a starting point to begin fully understanding the interactions between pea protein isolate and casein micelles combined via low-temperature homogenization and the effect on their techno-functional properties.

## Highlights

• Low-temperature homogenization was used to incorporate pea protein isolate with casein micelles.
• The blended protein system had similar rheology to fluid milk.
• The blended protein system retained the ability of caseins to coagulate upon rennet addition.
• The blended protein system had improved functional properties compared with a pea-protein-only system.

## Graphical Abstract

**Figure:**
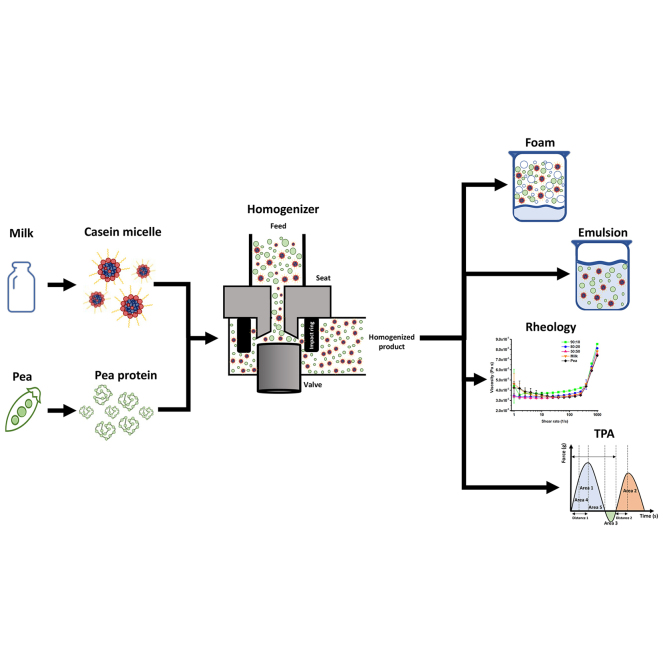
**Summary:** From a sample of skim milk, casein micelles were disrupted to expose their hydrophobic regions. Pea protein isolate was hydrated in water to create a pea protein slurry. Through cold-temperature homogenization, the insoluble pea proteins were incorporated with the casein micelles in a stable, mixed, colloidal dispersion. The foaming and emulsification properties of the blended protein system were measured and compared with pea-protein-only and milk-only solutions. The rheology of the blended protein system was measured and compared with the milk-only solution to determine the feasibility of dairy industry applications. Finally, the blended protein system underwent rennet coagulation; the resulting solid curd was texturally compared with a milk-only curd and a plant-based curd.

The market for plant-based protein has grown significantly in recent years as consumers seek more environmentally sustainable and ethically sound alternatives to animal-based protein. Pea protein (**PP**) is a pulse seed protein from yellow split pea (*Pisum sativum* L.) that has become popular due to its balanced AA composition and branched-chain AA that assist in muscle synthesis and expansion (Babault et al., 2015). Additionally, PP is a nonallergenic, gluten-free, and cost-effective plant-based protein. However, it can be a challenge to use in food systems because of its poor functionality, including low solubility and undesirable bitterness. Low solubility can hinder other functional properties of a protein, such as foaming, emulsification, gelation, and thickening (Fennema et al., 2008).

In recent studies, methods to improve the functionality of PP have been developed to increase the potential applications as a functional food ingredient. For example, Lan et al. (2019) was able to improve the solubility and flavor profile of PP through the use of solid dispersion–based spray drying. The beany off-flavors were masked through the unfolding of the PP secondary structure by forming solid dispersions with maltodextrin or gum arabic. Additionally, ultrasound was used to improve the solubility and emulsion properties of PP (O'Sullivan et al., 2016). Ultrasound treatment decreased the size of PP and decreased the droplet size of PP-stabilized emulsions. Finally, the partial replacement of plant-based proteins with animal protein has been investigated as a method to improve techno-functional properties (Alves and Tavares, 2019; Guyomarc'h et al., 2021). The process described in Krentz et al. (2022) fits in this category because it proposed the use of casein micelles to improve the solubility of hydrophobic PP. Low-temperature homogenization was used to facilitate hydrophobic interactions between caseins and PP to create a stable colloidal dispersion in an aqueous solution. A sample of skim milk with dissociated casein micelles was combined with a PP slurry. Sodium dodecyl sulfate-PAGE confirmed that the PP interacted with dissociated casein micelles, rather than with whey proteins (Krentz et al., 2022).

The protein functionality of caseins has been widely studied because of their importance in the food industry. Specifically, foaming and emulsification properties are especially critical in dairy products such as fluid milk, cream, whipped cream, ice cream, and butter. Caseins are widely used in the food industry to stabilize foams and emulsions due to their amphiphilic nature and ability to form a viscoelastic film at the water–air or water–oil interface (Fennema et al., 2008). Caseins are good emulsifiers because of their flexibility, lack of tertiary structure, and ability to quickly unfold and then reorient at the interface (Zayas, 1997). Additionally, caseins can form stable protein gels upon addition of rennet or acid. This property plays a crucial role in cheese manufacturing when separating the casein curd from the expelled liquid whey.

The objective of this study was to analyze the functional properties of casein micelle (**CM**):PP isolate blends created using the method described in Krentz et al. (2022). Specifically, the foaming, emulsification, texture, and rheology of the blends were analyzed to determine the efficacy of the blend to improve the functional properties of PP. We hypothesize that the CM:PP blend will have greater functional abilities than PP alone because of the incorporation of casein micelles in a colloidal dispersion in the aqueous phase.

To create the mixed protein blends, we followed the methodology described in Krentz et al. (2022). In brief, a milk sample (3.3% protein) was prepared by dissociating CM of commercial fat-free milk (Dean Foods Co.) with 5.16 g/L food-grade trisodium citrate (It's Just!, 138 Foods Inc.; Heertje et al., 1985). At the same time, a 2.6% (wt/vol) protein slurry was created with PP isolate (Mettle Nutrition LLC) and distilled water. The milk sample and the PP slurry were combined at various casein-to-PP ratios: 90:10, 80:20, and 50:50 (vol/vol). The samples were combined in a final volume of 30 L. Each protein blend underwent low-temperature (4°C) homogenization (APV 2-stage Homogenizer, P.M.S. Co.) followed directly by cooling (UHT/HTST Lab model 25HV Hybrid, MicroThermics Inc.). The blends were homogenized at 3,500 psi (24.1 MPa; first stage) and 500 psi (3.4 MPa; second stage) and fed directly into the UHT/HTST Lab Model 25HV Hybrid unit (MicroThermics) before the next cycle began. The blends were subjected to 3 continuous cycles of homogenization, immediately cooled, and recycled into the homogenizer. Subsequently, the batch was pasteurized at 63°C for 30 min. The sample blends were stored in sanitized 10-gallon milk cans at 4°C. Foamability and foam stability were measured using the mechanical mixing method described by Ho et al. (2019) with slight modifications. A 150-mL sample was poured into an electric milk frother (Bodum 11870 Bistro) and operated using the manufacturer's instructions. Foamability of the liquid CM:PP blends was determined as the ratio of foam volume (mL) to the volume of the initial liquid sample (mL). Foam stability (%) was determined by dividing the final foam volume (after 10 min) by the initial foam volume (at 0 min). Emulsion capacity and emulsion stability of the liquid CM:PP blends were measured using the methods described by Hung and Zayas (1991) with slight modifications, using a standing homogenizer (T25 Ultra Turrax model RN41, IKA) and commercial vegetable oil (Good and Gather). A 5-mL protein sample and 5 mL of vegetable oil were combined and mixed at 3,600 rpm for 5 min. The emulsion was transferred to a graduated cylinder and the volume of the serum phase measured after 30 min. Emulsion stability was measured via gravitational force. The calculation involved subtracting the initial serum phase volume from the final serum phase volume and dividing that difference by the initial serum phase volume. Emulsion capacity was determined as the ratio of the average amount of oil before and after the inversion point divided by the average amount of protein sample before and after the inversion point. A flow sweep was performed on a DHR-3 rheometer (TA Instruments) equipped with a DIN concentric cylinder, Peltier steel (115864). The protocol described by Chen et al. (2021) was followed with slight modifications. A steady flow sweep was performed with a shear rate range from 0.1 to 100 s^−1^ to determine the flow curves of the liquid CM:PP blends. The viscosity was plotted against the shear rate. Finally, the texture of the rennet-coagulated CM:PP curds was measured using a texture analyzer (TA-XTplusC, Stable Micro Systems). The CM:PP blends were coagulated using rennet based on the method described in Krentz et al. (2022). The parameters used followed those for the 2-bite analysis used by Li and Wang (2012) with slight modifications. Hardness, springiness, cohesion, and chewiness were measured from the compression curve. Commercial queso fresco and hard tofu were measured as controls. All experiments were conducted in triplicate. One-way ANOVA followed by Tukey-Kramer honestly significant difference (HSD) multiple comparisons were conducted to determine statistical differences (*P* < 0.05) between pairs using JMP software (JMP 14.0.0; SAS Institute Inc.).

The foamability and foam stability of the CM:PP blends were compared with that of skim milk and homogenized PP control, and results are shown in Figure 1a and b, respectively. Skim milk had the greatest foamability ratio of 2.41 ± 0.08, and the PP slurry had the lowest foamability ratio of 1.94 ± 0.10 (*P* = 0.01). All 3 CM:PP blends had similar foamability ratios (*P* = 0.08); however, they varied in their relation to the milk and PP slurry controls. The 90:10 and 80:20 blends had similar foamability ratios to milk (*P* = 0.90 and *P* = 0.36, respectively). The 80:20 and 50:50 blends had similar foamability ratios to the PP slurry (*P* = 0.14 and *P* = 0.98, respectively). As the amount of PP in the blends increased, the foamability ratio became closer to that of the PP slurry rather than milk. The incorporation of PP isolate with CM improved the foamability properties of the PP isolate but did not improve the foamability of CM. Based on the amount of PP added to the blended dairy- and plant-based system, product developers may create different products with foamability similar to that either of skim milk or of PP alone. The skim milk had similar foam stability (Figure 1b) as the 90:10 and 80:20 blends (*P* = 0.97 and *P* = 0.97, respectively). As the amount of PP in the blend increased to 50%, foam stability was closer to that of the PP slurry (*P* = 0.19). Foam stability is improved by greater protein concentration, which increases viscosity and protein film formation at the interface. The 90:10 blend had a higher protein content than the 50:50 blend due to mixing ratios and the additional whey protein present in milk. Similar to the foamability results, the mixed protein blends had better foam stability than PP alone. To fully understand the specific protein–protein interactions between CM and PP isolates, CM isolate should be used in future experiments to limit the effect of whey proteins and minerals present in skim milk.

**Figure 1:**
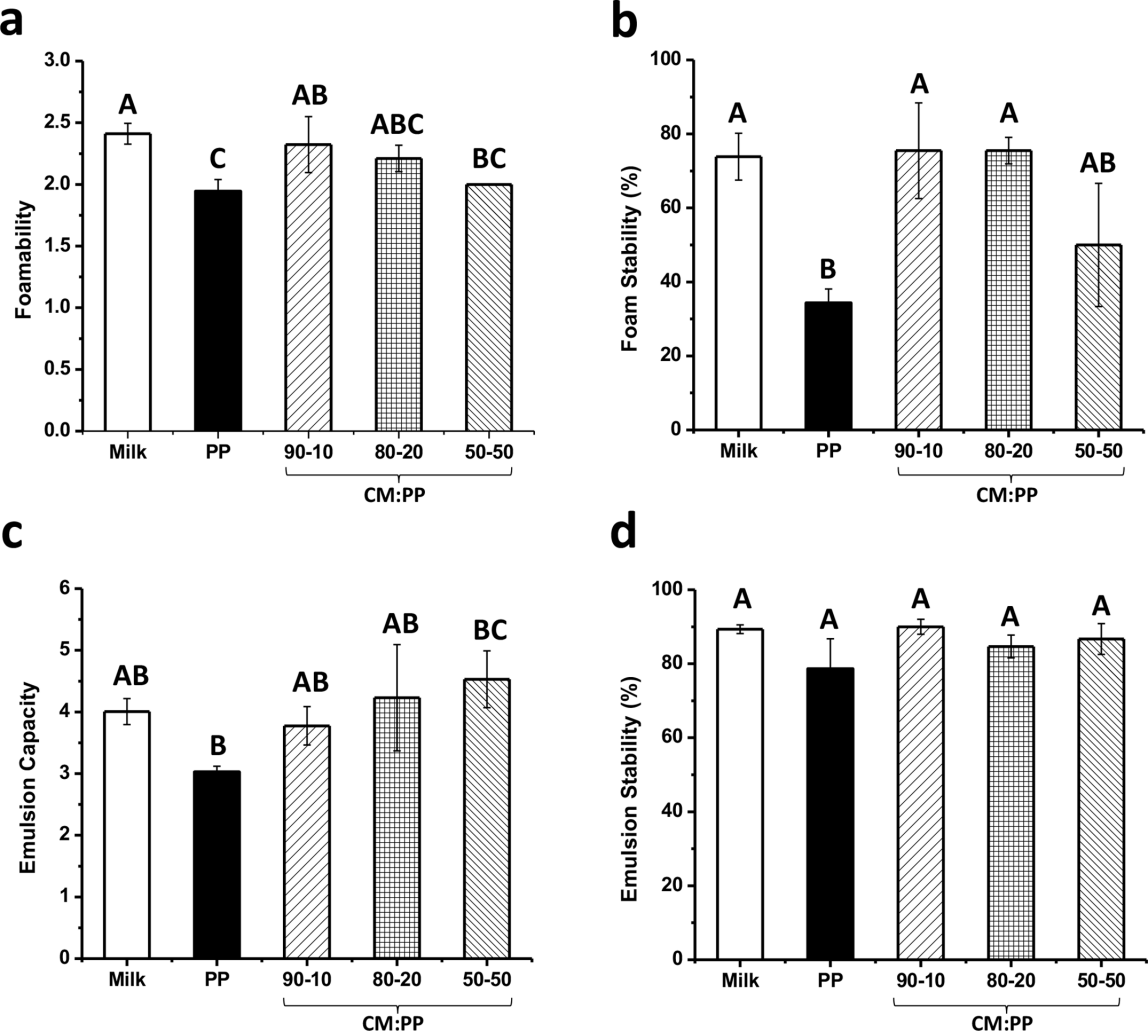
Foaming and emulsification properties of casein micelle:pea protein (CM:PP) liquid blends compared with milk and pea protein (PP) slurry controls: (a) foamability, (b) foam stability, (c) emulsion capacity, and (d) emulsion stability. Different letters (A–C) indicate significant differences between treatments (*P* < 0.05). Values are means of data from triplicate experiments; error bars indicate SD.

The emulsification capacity and stability were measured for each CM:PP blend and compared with that of skim milk and homogenized PP slurry (Figure 1c and d). The PP slurry had the lowest emulsion capacity, whereas the 50:50 CM:PP blend had the highest (*P* = 0.02). We expected that PP would have the lowest emulsion capacity because of the high percentage of globulin proteins, which are less flexible and take a longer time to orient at the water–oil interface (McClements, 2004). As the amount of PP in the blend increases, the emulsion capacity also increases. The ranking of the emulsion capacity of the CM:PP blends was as follows: 50:50 blend (4.53 ± 0.46) > 80:20 blend (4.23 ± 0.86) > 90:10 blend (3.77 ± 0.31). The 50:50 and 80:20 blends had higher emulsion capacities than skim milk (4.01 ± 0.21). These results indicate that the oil emulsion was w stabilized by the 50:50 CM:PP blend, at a higher capacity than either individual protein on their own. Ji et al. (2015) reported that the combination of the flexible animal protein and globular plant protein formed dense, mixed-protein, negatively charged layers around the oil droplet to successfully form a stable emulsion. The CM:PP blends had similar emulsion stability to both the milk and PP controls (*P* = 0.07). The amount of PP in the blend did not affect the stability of the emulsion (Figure 1d). This effect on emulsion stability may be due to the increase in solubility and the potential denaturation that may have occurred during homogenization that could have altered the PP structure to allow for better adsorption to the interface (Rampon et al., 2003; McClements, 2004). Additionally, the legumin-to-vicilin ratio may affect the emulsion properties of the PP slurry. Legumin and vicilin are the 2 main storage proteins in pea and have greatly differing structures that can affect functional properties. Isolated vicilin forms more stable emulsions than legumin, but isolated legumin has higher emulsion capacity (Liang and Tang, 2013). The commercial PP isolate used in these experiments may have had a greater percentage of vicilin than legumin. Further protein profile analysis of the PP isolate should be conducted to confirm this hypothesis.

The rheological properties of the CM:PP blends were compared with those of the milk and homogenized PP controls. We wanted to investigate the effect of the addition of PP on the rheology of the blended dairy- and plant-based solution to better understand how the product may behave during fluid flow in manufacturing. A flow sweep was conducted to monitor the changes of viscosity under different shear rates (Figure 2). At shear rates less than 100 s^−1^, the viscosity of the CM:PP blends, milk control, and homogenized PP control remained constant. Fluids that have a viscosity that is irrelevant to the applied shear rate, as long as constant temperature and pressure are maintained, are known as Newtonian fluids (Yahia et al., 2016). From shear rates of 1 to 100 s^−1^, all of our samples exhibited Newtonian fluid behavior. The viscosity of a solution is affected by the combination of short-range (repulsive) and long-range (attractive) intermolecular interactions between the components within the solution. Specifically, the viscosity of skim milk is largely dependent on the state and concentration of CM (Walstra and Jenness, 1984). For example, changes in CM (such as swelling), changes in pH, or decreases in temperature result in increased viscosity due to their increased volume. Despite the known increase in dispersed particle size in the CM:PP blends (Krentz et al., 2022), the incorporation of additional proteins in the milk system had a limited effect on the intermolecular interactions that affect viscosity. We hypothesize that the concentration of protein in the blended system (4%) was too low to have a significant effect on viscosity (Bienvenue et al., 2003). For example, Chen et al. (2021) observed that concentrated PP solutions (16%) showed shear thinning behavior rather than the Newtonian behavior of the 4% PP solution in this study.

**Figure 2:**
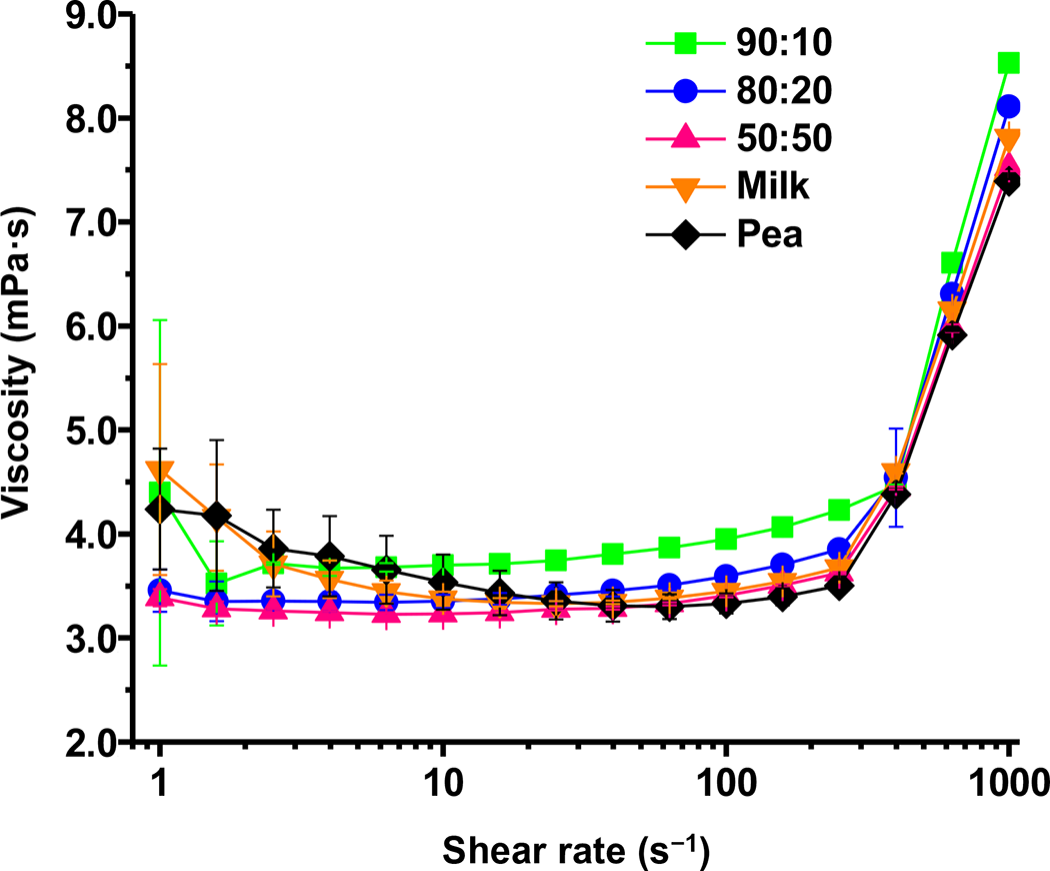
Typical flow curves of casein micelle:pea protein (CM:PP) liquid blends (90:10, 80:20, and 50:50) compared with milk and pea protein slurry controls. Values are means of data from triplicate experiments; error bars indicate SD.

Upon rennin coagulation, the CM:PP liquid blends formed a solid curd. The moisture and protein contents of the CM:PP curds were measured to further understand the textural properties. The ranking of the protein content of each CM:PP curd was as follows: 90:10 curd (28.22% ± 3.96) > 80:20 curd (28.00% ± 2.42) > 50:50 curd (19.36% ± 1.81). This ranking was as expected because the blends with higher ratios of CM:PP have the additional whey proteins in the milk contributing to the protein content. The ranking of the moisture content of each CM:PP curd was as follows: 90:10 curd (28.16% ± 3.08) > 80:20 curd (19.99% ± 3.87) > 50:50 curd (17.93% ± 3.63). We hypothesize that the CM have better water-holding capacity when less PP is present, due to less interference in the protein network. In general, the texture of milk curds is generally affected by protein, fat, and moisture contents (Chevanan et al., 2006; Yates and Drake, 2007).

The solid CM:PP curds were analyzed via a 2-bite analytical compression test to analyze the textural properties of the curd compared with commercial queso fresco and hard tofu (Figure 3). Queso fresco (Cacique) is a 100% milk-based fresh cheese with a milky and slightly salty flavor. Extra-firm tofu (Nasoya Foods) was chosen as the commercial 100% plant-based curd because it has a mild flavor and consumer-accepted texture for a bean-curd. The objective of this texture analysis was to determine how the different ratios of CM to PP affected the texture of the curds and how they compared with a 100% milk curd and 100% plant-based curd. Hardness is measured as the peak force of the first compression bite, and represents the force necessary to puncture the sample with human molar teeth (Chen et al., 1979). As the amount of PP in the blend increased (from 10% to 20% to 50%), the hardness significantly increased (Figure 3a). The ranking of the hardness of each CM:PP curd was as follows: 50:50 curd (182.38 ± 8.35) > 80:20 curd (89.33 ± 5.35) > 90:10 curd (43.83 ± 11.16). The hardness of the curd increased with decreasing moisture content. Hardness is negatively correlated with fat and NaCl and positively correlated with protein content (Chen et al., 1979). Additionally, the 50:50 curd had a finer internal structure because of the effects of PP on the internal structure of the cheese curd (Lee and Marshall, 1981); the finer structure resulted in a greater hardness. Regardless, the 90:10 curd and commercial tofu had similar hardness values (*P* = 1.00). Additionally, the 80:20 curd and commercial queso fresco had similar hardness values (*P* = 0.20). These results indicate the promising application of various CM:PP blends as curded products in the industry, because they have similar hardness levels to several consumer-accepted products. Figure 3b shows the springiness values of the CM:PP curds compared with the commercial products. Springiness is the rate at which a product returns to its deformed condition once the acting force is removed (Stable Microsystems texture analyzer test; https://www.stablemicrosystems.com/MeasureSpringiness.html). The 90:10 curd had a significantly higher springiness value than 80:20 and 50:50 curds (*P* = 0.001 and *P* = 0.03, respectively). In general, the samples with higher hardness levels and lower moisture contents had lower springiness levels. The 90:10 curd and commercial queso fresco had similar springiness values (*P* = 0.06). Figure 3c shows the cohesion values of the CM:PP curds compared with the commercial products. Cohesion is the measure of product deformation during compression before rupturing (Chen et al., 1979). The soy-based commercial tofu had a significantly greater cohesion value than the dairy products (*P* < 0.0001). Lee and Marshall (1981) found that the addition of soy proteins to coagulated milk curd decreased cohesiveness, as the fine divided structure was more susceptible to physical damage. We hypothesize that the addition of PP created a fine microstructure within the curd that was more susceptible to breakage. However, in this study, the 3 CM:PP curds had similar cohesion values to commercial queso fresco (*P* = 0.07), indicating that even if the incorporation of PP resulted in a finer microstructure, the cohesion would still be accepted by consumers. The last textural parameter analyzed was chewiness (Figure 3d), which is a measure of time needed to fully masticate the sample before swallowing (Chen et al., 1979). Chewiness is the product of hardness × springiness × cohesiveness (Chandra and Shamasundar, 2015). The rankings of chewiness values for the CM:PP curds were as follows: 50:50 curd (13.75 ± 3.82) > 90:10 curd (5.56 ± 0.27) > 80:20 curd (4.04 ± 1.35). The commercial tofu had a significantly greater chewiness value than all CM:PP curds (*P* < 0.0001). However, the commercial queso fresco had a similar chewiness value to the 50:50 curd (*P* = 0.36). It should be noted that chewiness is difficult to quantify precisely because of the multiple forces and lubrication needed to properly masticate a sample (Chandra and Shamasundar, 2015). In summary, the incorporation of PP within the casein curd affected the texture and structure of the curd. Different ratios of CM:PP can be applied in the blend to achieve the desired textural properties for specific food applications. Future experiments will involve acid coagulation and heat-induced coagulation to further understand the effect of PP incorporation on coagulation ability and curd structure.

**Figure 3:**
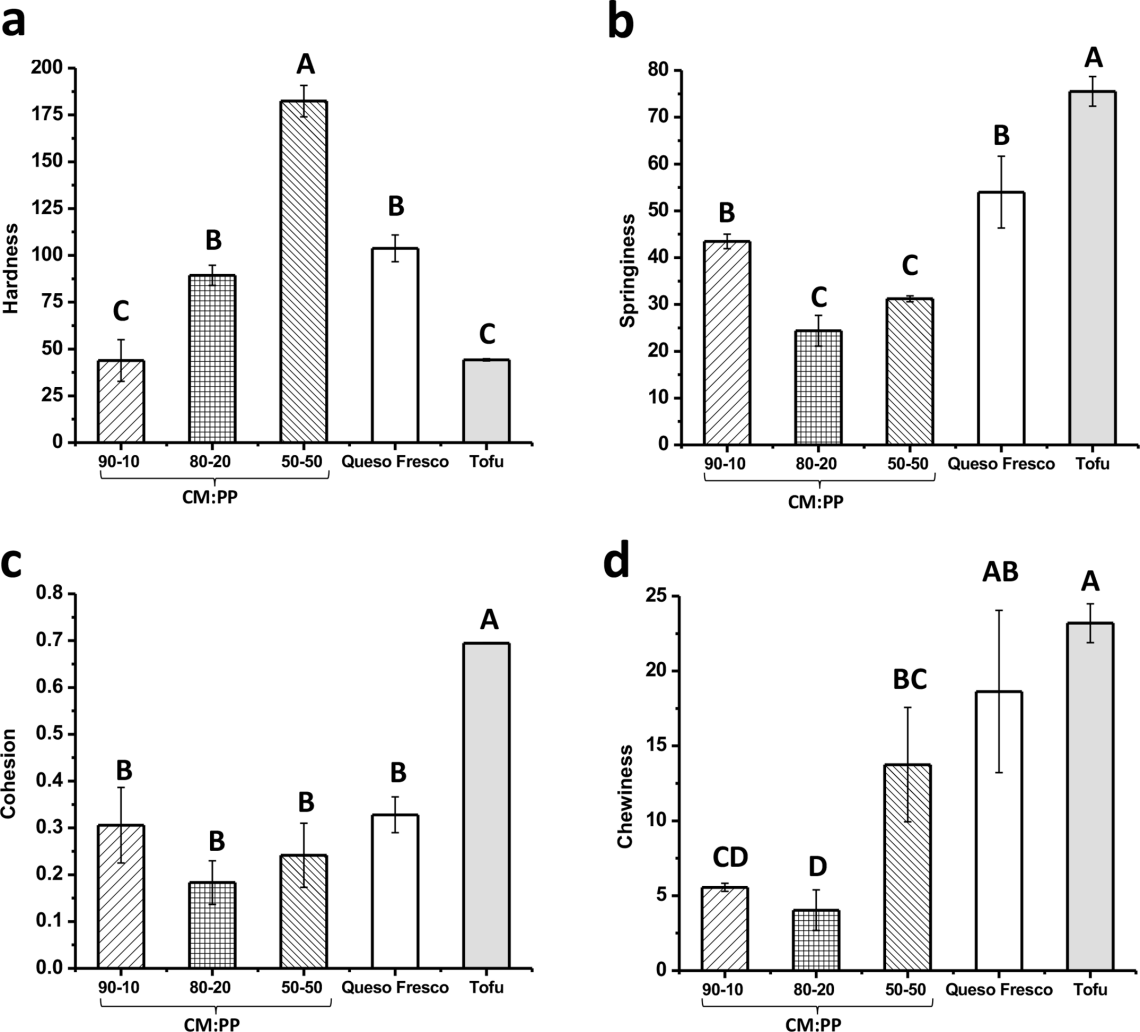
Texture profile analysis of casein micelle:pea protein (CM:PP) curds compared with commercial queso fresco and hard tofu: (a) hardness, (b) springiness, (c) cohesion, and (d) chewiness. Different letters (A–D) indicate significant differences between treatments (*P* < 0.05). Values are means of data from triplicate experiments; error bars indicate SD.

This work investigated the techno-functional properties of a mixed dairy and plant protein system created by the process described in Krentz et al. (2022). Krentz et al. (2022) determined that low-temperature homogenization between skim milk and a PP slurry resulted in increased solubility of PP in the aqueous phase and confirmed the interaction between PP and dissociated CM, where the CM were measured by dynamic light scattering, giving reliable results of the distribution of the particle size. The present work found that CM:PP blends had improved foaming and emulsification properties compared with the PP control. The mixed protein dispersion had a similar texture to commercial queso fresco and hard tofu, and similar fluid behavior to skim milk, providing multiple avenues for application of the CM:PP blends in either liquid or gel states. Future experiments should further investigate the interactions occurring between PP and CM on a molecular level. Better understanding of these interactions will assist in deciphering the changes seen in foaming and emulsification properties. This research highlights the potential to use CM to improve the functional properties of insoluble PP isolate.
